# Allopregnanolone and neuroinflammation: a focus on multiple sclerosis

**DOI:** 10.3389/fncel.2014.00134

**Published:** 2014-06-03

**Authors:** Farshid Noorbakhsh, Glen B. Baker, Christopher Power

**Affiliations:** ^1^Department of Immunology, Faculty of Medicine, Tehran University of Medical SciencesTehran, Iran; ^2^Department of Psychiatry, University of AlbertaEdmonton, AB, Canada; ^3^Department of Medicine (Neurology), University of AlbertaEdmonton, AB, Canada

**Keywords:** neurosteroid, allopregnanolone, neuroinflammation, multiple sclerosis, experimental autoimmune encephalomyelitis

## Abstract

The progesterone derivative allopregnanolone (ALLO) is one of the most widely studied compounds among neurosteroids. Through interactions with GABA-A receptors expressed by neurons and glial cells, ALLO has been shown to affect diverse aspects of neural cell physiology, including cell proliferation and survival, migration, and gene expression. Recent data point to important roles for ALLO in different neurodegenerative disorders, including Alzheimer's disease, Parkinson's disease, and multiple sclerosis (MS). Dysregulation in ALLO biosynthesis pathways has been reported in brain tissue from MS patients as well as in the central nervous system (CNS) tissue derived from MS animal models. Administration of ALLO has been shown to ameliorate neurobehavioral deficits together with neuropathology and inflammation in the CNS of animals with autoimmune demyelination. These findings are in line with previous reports indicating growth- and differentiation-promoting actions of ALLO on neurons and glial cells as well as its neuroprotective effects in the context of other CNS diseases. Nonetheless, these findings have also raised the possibility that ALLO might influence leukocyte biology and associated neuroinflammatory mechanisms independent of its neuroregenerative properties. Herein, we review the current knowledge regarding the role of ALLO in the pathogenesis of MS, and discuss the potential cellular and molecular pathways that might be influenced by ALLO in the context of disease.

## Introduction

Steroids synthesized from precursors in central or peripheral nervous systems have attracted substantial attention in recent years (Mellon and Griffin, 2002; Brinton, 2013). While the blood-brain barrier (BBB) is permissive to steroids produced by gonads and adrenals, the precise neuroanatomical segregation of the locally-synthesized steroids, i.e., neurosteroids, gives them a distinct advantage over peripheral steroids in terms of exerting region-specific effects in the nervous system. In addition to binding to intracellular steroid receptors, some neurosteroids interact with neurotransmitter receptors, thus influencing the biology of select types of neural cells (Belelli and Lambert, 2005). Reduction of locally-synthesized or peripherally-derived progesterone to dehydroprogesterone (DHP) and tetrahydroprogesterone (THP) is one of the widely studied arms of the neurosteroid synthesis pathway. While progesterone and DHP can both bind to intracellular steroid receptors, the predominant THP form, allopregnanolone, can only interact with and signal through cell surface GABA-A receptors.

Multiple sclerosis (MS) is a complex disease of the CNS with both inflammatory and degenerative aspects (Sospedra and Martin, 2005; Trapp and Nave, 2008). Neuropathologically, MS is characterized by leukocyte infiltration of CNS followed by myelin damage, local gliosis, and axonal injury. It is generally believed that the pathogenic process of MS is initiated by a breakdown of immune tolerance to CNS antigens due to genetic or environmental factors, leading to activation and proliferation of neuroantigen-reactive T cells in the peripheral immune system in susceptible individuals (Hemmer et al., 2002). Activated T cells and macrophages then infiltrate the CNS and become reactivated, leading to local microglial activation and intraparenchymal generation of chemokines and inflammatory cytokines. Subsequent waves of lymphocytic and monocytic cell infiltration into the CNS give rise to widespread neuroinflammation with ensuing myelin damage and axonal injury (Sospedra and Martin, 2005). Some evidence gives more weight to innate immune events, compared with adaptive immune processes, in MS neuroinflammation (Tsutsui et al., 2005; Mayo et al., 2012). An alternative view holds that initial neurodegenerative events, including apoptosis of oligodendrocytes or structural alteration of myelin, might occur in early stages of disease and then trigger subsequent inflammatory phenomena (Moscarello et al., 2007; Prineas and Parratt, 2012; Stys et al., 2012). Regardless of whether the initiating event in the MS disease process is an innate or adaptive immune dysregulation or a neurodegenerative/cell death phenomenon, destruction of myelin and axonal damage are the final pathogenic outcomes which underlie signs and symptoms of the disease.

The localized and well-demarcated nature of pathology in MS begs the question of whether a focal dysregulation in the CNS microenvironment might contribute to the disease process in addition to the infiltration of myelin-reactive T cells and other leukocytes. Numerous studies have shown the link between MS with steroid hormones (El-Etr et al., 2005; Simpkins et al., 2005; Kipp and Beyer, 2009). Following the discovery of neurosteroids in the CNS, efforts were made to elucidate the role of these compounds and their potential dysregulation in the context of MS and neuroinflammation. Herein, we review the current knowledge regarding the role of ALLO in the MS disease process. The first part of this review concentrates on the effects of ALLO on major cellular players in MS pathogenesis, i.e., oligodendrocytes, monocytoid cells, and lymphocytes. While direct effects of ALLO on neuronal physiology might also be important in the context of MS and in particular on the degree of axonal injury which is correlated with MS symptoms, we have not included the neuronal effects of ALLO here and we refer the interested reader to other reviews on this subject (Charalampopoulos et al., 2006, 2008; Leskiewicz et al., 2006). In the second part of the review, we assess the evidence supporting the involvement of ALLO in MS pathogenesis, including studies in animal models of disease, as well as its potential for therapeutic interventions.

## ALLO and MS pathogenesis; cellular players

### ALLO, oligodendrocytes, and myelination

The effects of progesterone and its derivatives in promoting myelin formation in the peripheral nervous system (PNS) have been recognized for almost two decades (Baulieu and Schumacher, 1997). Schwann cells have been shown to express functional GABA-A receptors and respond to ALLO treatment by upregulating two major myelin proteins, i.e., myelin protein 22 and P0 (Melcangi et al., 1999). Progesterone and ALLO were later reported to induce myelin basic protein (MBP) gene expression in brain organotypic slice cultures (Ghoumari et al., 2003). While the effect of progesterone on MBP gene expression was shown to be largely mediated by intracellular progesterone receptors, effects of ALLO were mediated by GABA-A receptors expressed on oligodendrocytes (Ghoumari et al., 2003). In addition to promoting myelin protein gene expression in mature oligodendrocytes, progesterone has been illustrated to enhance the proliferation of oligodendrocyte precursors in cerebellar slice cultures through intracellular progesterone receptors, but a similar effect has not been reported for ALLO (Ghoumari et al., 2005). That said, ALLO has been shown to induce proliferation of rat hippocampal neuroprogenitor cells or human cortical neural stem cells, with the resulting cells showing neuronal phenotype (Wang et al., 2005). In addition to its promyelinating effects, ALLO has been reported to protect oligodendrocytes against cytotoxic stimuli. When treated with recombinant TNF-α, cultured rat oligodendrocytes display reduced viability as quantified by CNPase immunoreactivity. Pretreatment with ALLO (100 nM) reduced the toxicity of TNF-α (Noorbakhsh et al., 2011). Overall, it seems that ALLO exerts promyelinating as well as cytoprotective effects on oligodendrocytes against inflammatory stimuli, and both effects are relevant because of the beneficial outcomes in the context of autoimmune demyelination.

### ALLO and monocytoid cells

Macrophages are known to express functional GABA-A receptors, and the activation of the receptors leads to reduced production of inflammatory cytokines by these cells (Reyes-Garcia et al., 2007). Treatment with different GABA-A agonists have been shown to alter the behavior of macrophages and dendritic cells and the resulting T cell response following antigen presentation (Bhat et al., 2010). When treated with ALLO, murine peritoneal macrophages have been demonstrated to produce lower levels of TNFα after LPS stimulation (Ghezzi et al., 2000). In a study from our group, treatment of human monocyte-derived macrophages with ALLO (100 nM) reduced the production of IL-1β and TNF-α transcripts after PMA exposure. ALLO treatment also reduced macrophage expression of IDO, an enzyme involved in a variety of inflammatory processes (Noorbakhsh et al., 2011).

In addition to monocyte infiltration of the CNS, activation of microglia, the resident monocytoid cells of the brain, also plays an critical role during neuroinflammatory processes (Jack et al., 2005). Similar to macrophages, microglia have been illustrated to express both GABA-A and GABA-B receptors, and treatment with the GABA-A agonist muscimol reduces microglial production of inflammatory mediators following LPS stimulation (Lee et al., 2011). Nonetheless, studies investigating the effect of ALLO on microglial function are limited. A study by Muller et al. has shown decreased production of NO by LPS-stimulated microglial BV2 cell lines after treatment with either progesterone or ALLO (Muller and Kerschbaum, 2006). While still an underappreciated area, the consequences of microglial GABA-A signaling might not be limited to reduced proinflammatory activity of these cells. An interesting study by Mead et al. has shown that activation of GABA-A receptors on microglia can lead to enhanced activity of different isoforms of NADPH oxidase (Nox), the enzyme responsible for generation of superoxide ions (Mead et al., 2012). While Nox activation by glutamate signaling leads to a neurotoxic phenotype, GABA-A-receptor-mediated activation of the enzyme promotes a neuroprotective phenotype (Mead et al., 2012). It remains to be explored if ALLO-mediated activation of microglial GABA-A receptors might contribute to differentiation of the cells toward a neuroprotective anti-inflammatory phenotype.

### ALLO and lymphocytes

While altered activity of antigen presenting cells after exposure to ALLO or other GABA-A receptor agonists could translate into alterations in lymphocyte proliferation and differentiation, little is known about direct effects of ALLO on lymphocytes. Human and murine lymphocytes have been shown to express functional GABA-A receptors (Mendu et al., 2012). Treatment with the GABA-A agonist muscimol have been shown to inhibit antigen-specific T cell proliferation (Tian et al., 1999) and GABA-A activation leads to whole-cell transient and tonic currents in T lymphocytes (Mendu et al., 2012). Nonetheless, in a study by our group, ALLO treatment of splenocyte cultures derived from animals immunized with a myelin antigen did not affect cell proliferation after antigenic re-stimulation (Noorbakhsh et al., 2011). Moreover, ALLO treatment did not affect differentiation of antigen-stimulated lymphocytes to Th1 or Th17 pathogenic phenotypes, as measured by intracellular immunostaining of IFN-γ and IL-17, the prototypic Th1/Th17 cytokines (Noorbakhsh et al., 2011). While these findings do not rule out the possibility of ALLO acting directly on lymphocytes, they are more supportive of the role of ALLO as a modulator of innate immune function.

### ALLO and leukocyte migration through blood brain barrier (BBB)

A critical step in the process of autoimmune neuroinflammation is the traversing of the BBB by peripherally-activated leukocytes. These cells include neuroantigen-reactive lymphocytes or monocytes entering the CNS following the chemokine gradient generated by locally activated microglia or previously infiltrated lymphocytes. Both progesterone and ALLO have been demonstrated to reduce BBB dysfunction following focal ischemia (Ishrat et al., 2010). This effect has been partly attributed to suppressed expression of MMP-2 and MMP-9 in ischemic brain following ALLO treatment (Ishrat et al., 2010). Moreover, ALLO was shown to prevent degradation of the BBB tight junction proteins occludin 1 and claudin 5 (Ishrat et al., 2010). While it remains to be investigated, it is conceivable that ALLO treatment could also affect BBB permeability and leukocyte trafficking during autoimmune inflammation.

**Figure 1:**
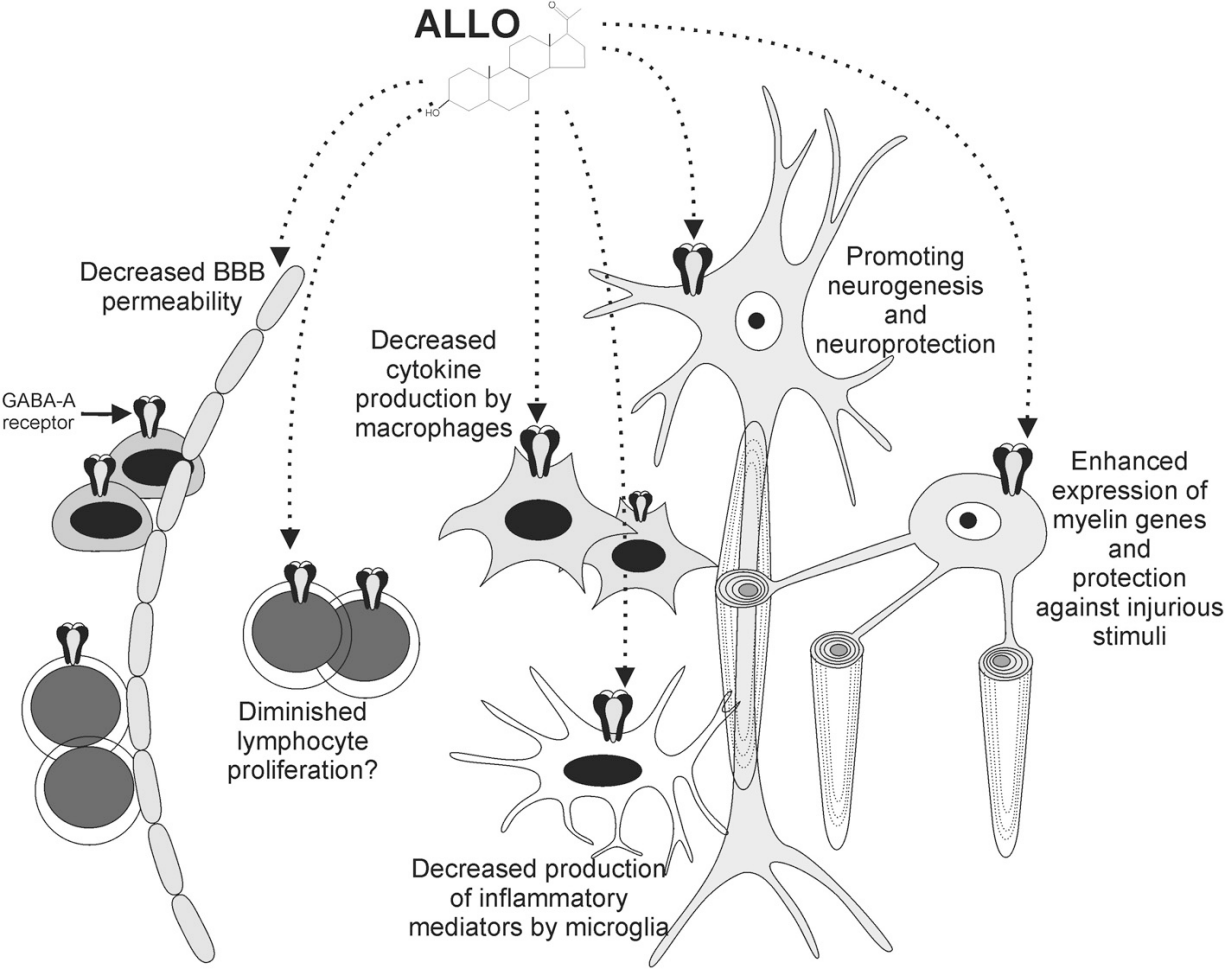
**ALLO exerts various effects on cells involved in MS pathogenesis**. Functional GABA-A receptors are expressed by neurons, oligodendrocytes, monocytoid cells, and lymphocytes. ALLO promotes myelin gene expression by oligodendrocytes and protects them against injurious stimuli. Neuroprotective effects have also been reported for neurons. ALLO's binding to GABA-A receptors on monocytoid cells leads to diminished production of inflammatory mediators by these cells. These effects, together with diminished BBB permeability and potential influence on lymphocytes, contribute to the beneficial roles of ALLO in the context of autoimmune demyelination.

We have summarized the effects of ALLO on different cellular elements with known roles in MS pathogenesis (Figure 1).

## ALLO and MS pathogenesis, human studies and animal models

The role of progesterone and its potential derivatives in demyelinating diseases has been studied for many years (Shuster, 2008). Before the identification of neurosteroid synthesis pathways in the brain, it had been reported that treatment of mice with experimental autoimmune encephalomyelitis (EAE) with progestins ameliorated disease severity (Arnason and Richman, 1969a,b). Later studies further highlighted the protective and anti-inflammatory role of progesterone in EAE (Garay et al., 2007). Progesterone treatment of neuroantigen-reactive CD4+ T cells isolated from MS patients was shown to affect their cytokine production (Correale et al., 1998). Progesterone and its reduced form dehydroprogesterone (DHP) can bind to intracellular progesterone receptors and exert genomic effects via altered expression of progesterone-responsive genes. However, ALLO lacks this ability and can only exert its effects through interactions with GABA-A receptors (Paul and Purdy, 1992). Considering that exogenously-administered progesterone could be readily reduced to ALLO in different cells, the protective effects reported for progesterone have likely been the consequence of both genomic regulation and GABA-A receptor-mediated pathways.

Subsequent studies provided more specific evidence supporting the role of ALLO in MS pathogenesis. In a study performed on human autopsy brain tissues, our group showed that ALLO levels were significantly reduced in the brain white matter derived from MS patients compared with control individuals (Noorbakhsh et al., 2011). The reduction was associated with diminished levels of two crucial upstream enzymes which are involved in conversion of progesterone to ALLO, i.e., 5-α-reductase and AKR1C1/C2, in brain white matter of MS patients. The levels of the further upstream enzyme, 3-α-HSD, which is involved in conversion of pregnenolone to progesterone, was not altered in MS brains. Interestingly, analysis of the levels of 3α,5β-THP, a minor isoform of THP, did not show any changes in the same brain tissues, indicating a specific downregulation in 3α,5α-ALLO isoform. Similar analysis of spinal cord tissues derived from EAE mice showed decreased levels of ALLO, but not the minor THP isoforms in EAE mice compared with control animals (Noorbakhsh et al., 2011). Again, ALLO suppression was associated with diminished RNA and protein levels of murine isoforms of the AKR1 enzymes, akr1c14, and akr1e1. Treatment of EAE mice with daily intraperitoneal injections of ALLO, which were started after the onset of neurological signs, reduced disease severity. Neuropathological analyses of the spinal cords from the same animals revealed diminished myelin damage and axonal injury in ALLO-treated animals, a finding that was consistent with the clinical features. These observations were also associated with lower levels of lymphocyte infiltration and monocyte/microglial activation in the CNS (Noorbakhsh et al., 2011).

Enhanced synthesis of endogenous ALLO has also been reported to be protective in the context of autoimmune demyelination. Translocator protein (TSPO) is a transfer protein located in the outer membrane of mitochondria (Papadopoulos et al., 2006; Gatliff and Campanella, 2012) previously termed the peripheral benzodiazepine receptor and a recognized marker for activated glial cells (Venneti et al., 2006). Through interactions with StAR (steroidogenic acute regulatory protein), TSPO exerts a rate-limiting step in the transfer of cholesterol into mitochondria, where it can be converted to pregnenolone (Rone et al., 2009). It has been shown that modulation of TSPO activity with etifoxine, a TSPO ligand, can enhance neurosteroid synthesis in brain (Girard et al., 2012; Nothdurfter et al., 2012). A recent study has shown that modulation of TSPO activity with etifoxine ameliorates disease symptoms and neuropathology in mice affected with EAE (Daugherty et al., 2013). The treatment was effective when the drug was administered at the presymptomatic stage of disease or at the peak of disease. The protective effect of TSPO activation was shown to be associated with specific upregulation of the murine enzyme responsible for ALLO synthesis, i.e., akr1c14 (Daugherty et al., 2013). Other TSPO ligands have also been reported to diminish microglial activation, an effect that is likely mediated through enhanced microglial neurosteroid synthesis (Zhao et al., 2011; Leaver et al., 2012). Of note, TSPO shows upregulation on astrocytes and microglia in a variety of neuroinflammatory disorders, which might be a part of an endogenously-regulated protective response in the context of neuroinflammation (Abourbeh et al., 2012; Lavisse et al., 2012).

## Future perspectives: ALLO as a therapy for MS

Since the discovery of ALLO dysregulation in several neurological disorders, ALLO and its synthetic analogs have been considered for their potential therapeutic or disease modifying effects in brain disease (Brinton, 2013). Currently, the majority of evidence supporting a beneficial role for ALLO and its analogs comes from animal models, with few studies having tested the effects of ALLO treatment on humans. In a pioneering study by Griffin et al., ALLO was shown to diminish neurological signs and neuropathology in a mouse model of Niemann-Pick disease (Griffin et al., 2004). ALLO treatment was later shown to decrease beta-amyloid burden and memory deficits in triple-transgenic mouse models of Alzheimer's disease (Wang et al., 2010; Singh et al., 2012), restore dopaminergic neurons and motor performance in the MPTP model of Parkinson's disease (Adeosun et al., 2012), exert anticonvulsant activity and decrease neuronal injury in models of epilepsy (Mares et al., 2006, 2010; Singh et al., 2010), reduce infarct volume and improve cognitive outcome in models of brain ischemia (Sayeed et al., 2006; Morali et al., 2011), and reduce neuronal death and gliosis after traumatic brain injury (TBI) (Djebaili et al., 2005). Of note, the protective effects of ALLO in TBI have been clearly linked with its anti-inflammatory functions (He et al., 2004; VanLandingham et al., 2007). Evidence for potential therapeutic roles for ALLO in MS also comes from animal studies showing reduced neuroinflammation and disease burden in the EAE animal model of disease after treatment with ALLO or TSO ligands, which lead to induction of ALLO-synthesizing enzymes (Noorbakhsh et al., 2011; Daugherty et al., 2013). While several reports have demonstrated the safety of ALLO administration to humans (Timby et al., 2006; van Broekhoven et al., 2007), ALLO trials on human disease are limited, in part because of costs and the quantity of drug needed for therapeutic purposes. Of interest, ganaxolone, an ALLO synthetic analog, has been successfully used to control epilepsy in human cases (Nohria and Giller, 2007; Pieribone et al., 2007), and recent studies from our group indicate that ganaxolone also exerts anti-inflammatory effects in EAE (Paul et al., 2014). Clinical trials to evaluate the therapeutic effects of ALLO in TBI are currently underway (Clinicaltrials.gov, NCT01673828), while ALLO trials for treating mild cognitive impairment and Alzheimer's disease are also expected (Irwin and Brinton, 2014). Overall, considering the wealth of knowledge derived from *in vitro* and *in vivo* analyses, together with low toxicity and good tolerance in human subjects, ALLO and its analogs are excellent candidates waiting to be tested in MS and other neuroinflammatory disorders.

### Conflict of interest statement

The authors declare that the research was conducted in the absence of any commercial or financial relationships that could be construed as a potential conflict of interest.
